# Inter-personal diversity and temporal dynamics of dental, tongue, and salivary microbiota in the healthy oral cavity

**DOI:** 10.1038/s41522-016-0011-0

**Published:** 2017-01-26

**Authors:** Michael W. Hall, Natasha Singh, Kester F. Ng, David K. Lam, Michael B. Goldberg, Howard C. Tenenbaum, Josh D. Neufeld, Robert G. Beiko, Dilani B. Senadheera

**Affiliations:** 1 Faculty of Computer Science, Dalhousie University, Halifax, NS Canada; 2 Faculty of Dentistry, University of Toronto, Toronto, ON Canada; 3 Department of Biology, University of Waterloo, Waterloo, ON Canada; 4 Department of Dentistry, Division of Research, Mount Sinai Hospital, Toronto, Ontario Canada; 5 School of Dental Medicine, Tel Aviv University, Tel Aviv, Israel

## Abstract

Oral microbes form a complex and dynamic biofilm community, which is subjected to daily host and environmental challenges. Dysbiosis of the oral biofilm is correlated with local and distal infections and postulating a baseline for the healthy core oral microbiota provides an opportunity to examine such shifts during the onset and recurrence of disease. Here we quantified the daily, weekly, and monthly variability of the oral microbiome by sequencing the largest oral microbiota time-series to date, covering multiple oral sites in ten healthy individuals. Temporal dynamics of salivary, dental, and tongue consortia were examined by high-throughput 16S rRNA gene sequencing over 90 days, with four individuals sampled additionally 1 year later. Distinct communities were observed between dental, tongue, and salivary samples, with high levels of similarity observed between the tongue and salivary communities. Twenty-six core OTUs that classified within *Streptococcus, Fusobacterium, Haemophilus, Neisseria, Prevotella*, and *Rothia* genera were present in ≥95% samples and accounted for ~65% of the total sequence data. Phylogenetic diversity varied from person to person, but remained relatively stable within individuals over time compared to inter-individual variation. In contrast, the composition of rare microorganisms was highly variable over time, within most individuals. Using machine learning, an individual's oral microbial assemblage could be correctly assigned to them with 88–97% accuracy, depending on the sample site; 83% of samples taken a year after initial sampling could be confidently traced back to the source subject.

## Peering into the mouth: Bacterial turnover in plaque and saliva

A study of bacteria in the mouth reveals insights into their diversity, stability, and variability among people and over time. By tracking daily, weekly, and monthly fluctuations of plaque and salivary bacteria in ten healthy volunteers, Dilani Senadheera at the Faculty of Dentistry, University of Toronto and co-researchers in Canada reveal significant differences in the “microbiome” present in dental, tongue and saliva samples over time. They found considerable variation in these communities between individuals, sufficient to identify a person with “bacterial fingerprints” using plaque or saliva even after 1 year. The researchers reveal a “core community” that spans different persons, oral sites, and time, suggesting some level of stability. This study is useful to understand the diversity and community drifts in different oral sites over time, which is important when plaque and saliva are used for bacterial analysis in diagnostic, risk-prediction, and forensic applications.

## Introduction

The resident microbial consortia present in the mouth, gut, skin, nasal cavity, and urogenital tract are intimately associated with human physiological functions including immunity, metabolism, and nutrition.^1^ The oral cavity acts as the primary portal of entry to the human digestive tract and the oral biofilm harbors one of the most microbiologically diverse sites within the human body.^2,3^ The mouth contains hard non-shedding tooth surfaces, which allow for microbial colonization and formation of the dental plaque biofilm. In addition to teeth, the mouth contains a wide variety of habitats including the tongue, gingival sulcus, cheek, and both hard and soft palates that contribute to its vast ecological complexity.^4^ These sites create niches that are influenced by variations in oxygen and nutrient availability, mechanical stress, and salivary flow, which together support the colonization and sustenance of distinct communities.^4^ The oral environment can differ between subjects as a result of the quality and quantity of saliva, variability in social habits (e.g., tobacco intake, diet, exposure to medications/dentifrices/antimicrobial mouth rinses), hormonal fluctuation, and variability in levels of host defense mechanisms.^4^ These behavioral, environmental, and genetic factors may impact the oral microbiota by influencing various ecological parameters. Dysbiosis of the microbial communities in the mouth is associated with oral infections including dental caries and periodontitis.^5,6^ Hence, an in-depth understanding of the baseline fluctuations for the healthy core oral microbiota presents an opportunity to examine their shift during the onset and recurrence of disease.

In spite of the diversity and dynamism of oral habitats, knowledge pertaining to the temporal stability of the oral microbiota remains limited. To date, studies considering temporal influences on oral microbiota have focused on either a single habitat in the oral cavity^7–12^ or have focused on a limited set of time points or duration of study.^13–15^ However, an in-depth investigation involving more sampling time points and multiple habitats is necessary to elucidate the core microbiota and identify interrelated community connections between different oral habitats. The utility of oral microbial biomarkers in translational applications necessitates our understanding of the daily, weekly, and monthly dynamics of microbial communities in different oral sites, when a single sampling time-point is commonly used for risk-assessment and prognostic approaches. Although dynamics of the oral microbiome has been examined as part of larger studies examining microbiota of the human digestive tract, to date there are relatively few longitudinal studies that have focused exclusively on temporal shifts of the oral microbiome. Findings from other studies which examined the tongue and supragingival sites in up to 85 people over 3 months, and the saliva in two subjects over 1 year, suggest that the bacterial composition in these habitats varies temporally.^9,16^ In contrast, the current paradigm suggests that the core plaque microbiota is in a state of microbial homeostasis^4^; some studies indicate that the oral microbiota remain stable over time, at least in healthy subjects.^7,10,17–19^ Temporal oral microbiota patterns may depend on the time scale. For example, the core microbiota can fluctuate on time scales of 48–72 h, with stability observed over longer periods of time.^9^

Here we explored the temporal dynamics of the oral microbiota by characterizing 286 microbial samples obtained on daily, weekly, and monthly intervals in ten healthy individuals over a study period of 90 days, with four individuals revisited after 1 year. Using high-throughput sequencing, we identified both stable and variable components of the healthy oral microbiota in saliva, supragingival, and tongue plaque within this time frame. For example, community composition was often variable, especially that of rare microorganisms. In contrast, we identified a stable core set of microorganisms that was shared between all time points, oral sites, and individuals and accounted for a large proportion of the sequence data. Community diversity was variable between individuals, but remained consistent within an individual over time.

## Results

To investigate the longitudinal stability and characterize intra—and inter-individual variability of the plaque and salivary microbiota, 286 samples containing supragingival plaque, tongue dorsum plaque, and saliva, were obtained from ten healthy volunteers over a period of 90 days (sampled on days 1, 2, 3, 4, 7, 14, 21, 28, 60, and 90). Four of the subjects were sampled at an additional time point after 365 days. After paired-end assembly, quality filtering, chimera removal, and 97% operational taxonomic unit (OTU) clustering, 12,097,786 sequences were clustered into 1805 OTUs with an average of 42,300 (±19,189 sd), a minimum of 8761, and maximum of 117,179 sequences per sample. The OTU table was evenly subsampled to 8761 sequences per sample. At this sequencing depth, supragingival and tongue sites harbored an average of 223 and 248 OTUs, respectively; saliva contained an average of 291 OTUs.

### Supragingival, tongue, and salivary communities display strong inter-individual and inter-site differences

**Fig. 1:**
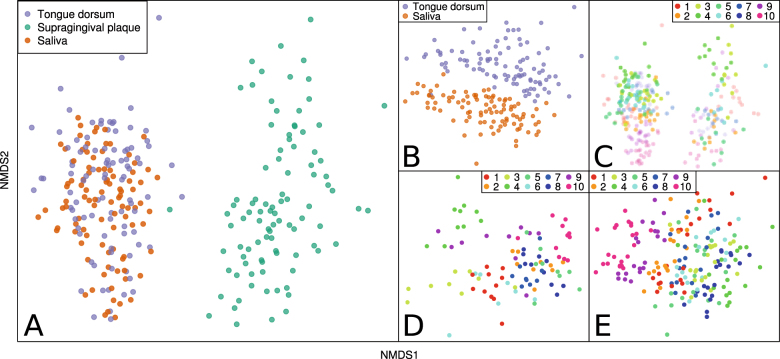
NMDS ordinations of samples showing sample dissimilarity patterns under the Bray–Curtis measure. **a** All samples *colored* by oral site (stress value: 0.16). **b** Tongue and saliva samples *colored* by oral site (stress value: 0.20). **c** All samples *colored* by subject number (stress value: 0.16). **d** Supragingival plaque samples *colored* by subject number (stress value: 0.16). **e** Tongue and saliva samples *colored* by subject number (stress value: 0.20)

Non-metric multidimensional scaling (NMDS) ordinations based on Bray–Curtis dissimilarities highlight clear patterns in community composition (Fig. 1). The multi-response permutation procedure (MRPP) was used to assess significance and effect size of group separation which is quantified by the *A* value, where a higher *A* value means better within-group agreement and between-group separation. Tongue plaque and saliva communities cluster together distinctly from supragingival plaque communities (Fig. 1a, MRPP: *p* < 0.001, *A* = 0.15). When supragingival plaque samples are excluded from the analysis, tongue plaque, and saliva communities show clear differences, but with a much smaller effect size (Fig. 1b, MRPP: *p* < 0.001, *A* = 0.02). Communities from a single subject cluster closely to one another whether considering all sites at once (Fig. 1c, MRPP: *p* < 0.001, *A* = 0.16), supragingival plaque (Fig. 1d, MRPP: *p* < 0.001, *A* = 0.35), or saliva and tongue plaque together (Fig. 1e, MRPP: *p* < 0.001, *A* = 0.26).

### Phylogenetic diversity is consistent between subjects in tongue plaque, and variable in supragingival plaque and saliva

**Fig. 2:**
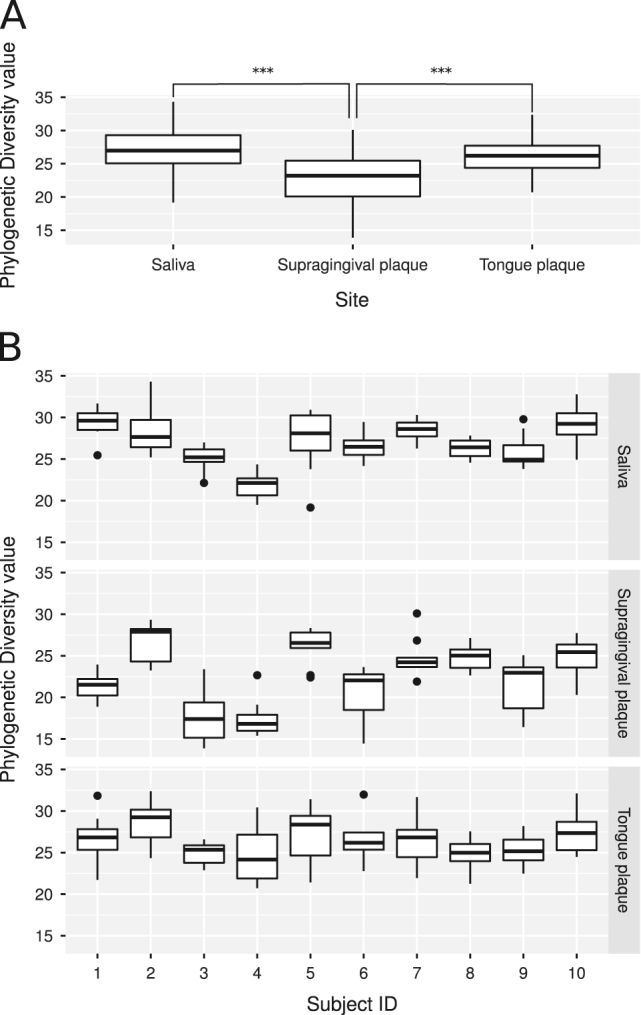
*Boxplots* of phylogenetic diversity measures. **a** PD values grouped by sample site (*** = significant at *p* < 0.001 by pairwise Wilcox test with false discovery rate correction). **b** PD values grouped by sample site and subject

We measured alpha diversity using Faith’s phylogenetic diversity (PD) measure.^20^ Supragingival plaque samples had significantly lower PD compared with that of tongue and salivary communities (Fig. 2a; *p* < 0.001 by pairwise Wilcoxon test with false-discovery-rate correction). The number of observed OTUs differed significantly between all three sites. The highest number of OTUs was observed in saliva, whereas supragingival plaque harbored the lowest OTU number (data not shown). Also notably, in contrast to saliva and supragingival samples that showed significant differences in PD between subjects, no significant differences were observed between subjects across tongue samples (Fig. 2b). In particular, subjects 3 and 4 had lower supragingival and salivary PD values than most of the other subjects. Heterogeneous variance was observed across the sample sites (*p* < 0.01 by Fligner-Killeen test), with significantly higher variance in PD values in supragingival plaque compared with tongue plaque and saliva. Within each site, the PD values showed similar levels of variation from subject to subject (*p* > 0.05 by the Fligner-Killeen test).

### Each individual harbors a distinct oral bacterial community fingerprint as verified by machine learning

Random-forest classification was used to help quantify the uniqueness of the personal oral community structure. We trained the classifier on random subsets of samples using the OTUs as features. The subject identifier was predicted from samples that were held back from the training set. With all samples pooled, random-forest classification achieved an accuracy of 95% (±3.8). Using only samples from within each site, the subjects were predicted with accuracies of 96.6% (±3.2) for saliva, 96.0% (±4.4) for supragingival plaque, and 88.2% (±7.9) for tongue plaque. We trained an additional random-forest classifier against all of the samples collected in the first 90 days, and predicted the source subject of the 12 samples taken at 365 days. All three sites pooled together had a prediction accuracy of 83.3%, supragingival plaque had an accuracy of 75% (one misclassification), whereas tongue plaque and saliva samples achieved 100% accuracy.

### Twenty-six core OTUs persist across sites, individuals, and time

**Fig. 3:**
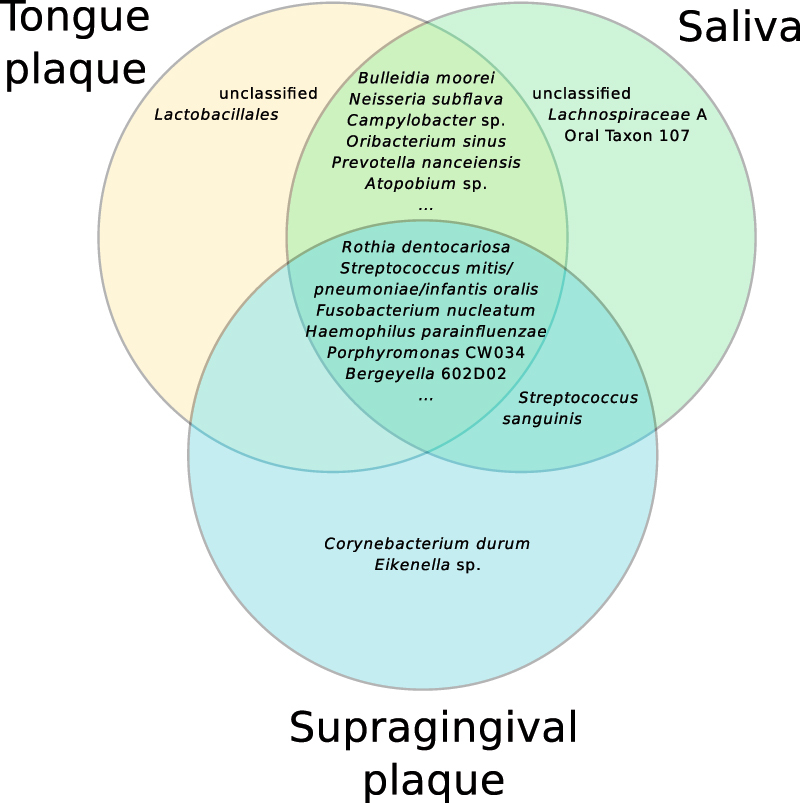
Venn diagram showing the taxonomic classifications of some of the core OTUs present within ≥95% of the samples in the indicated subsets of oral sites. For example, *Bulleidia moorei* was found in ≥95% of tongue plaque and saliva samples, but <95% of supragingival plaque samples, while *Rothia dentocariosa* was found in ≥95% of the samples from all three sites. This list is not exhaustive; see Supplementary File 1 for a complete list

In order to highlight the most consistent members of the oral microbiota, we identified “core”^21,22^ OTUs that were present in ≥95% of all collected samples. Twenty-six OTUs were present nearly universally in all three sites, ten subjects, and time points (Supplementary File 1). The core OTUs belonged to five phyla: *Actinobacteria, Bacteroidetes, Firmicutes, Fusobacterium,* and *Proteobacteria*. Core OTUs identified down to the species level included: *Rothia dentocariosa*, *Rothia mucilaginosa,* *Actinomyces odontolyticus*/*lingnae* *, Actinomyces viscosus/naeslundii/oris, Porphyromonas* clone CW034, *Prevotella melaninogenica, Bergeyella* clone 602D02*, Streptococcus mitis/pneumoniae/infantis/oralis, Neisseria subflava,* and *Haemophilus parainfluenzae*. Eight of the twenty-six core OTUs could not be classified to genus level, with one OTU unclassified at the phylum level. The twenty-six core OTUs accounted for approximately 65% of the sequences in the data. Site-specific core OTUs were identified by requiring presence in ≥95% of samples from a given oral site (Fig. 3; Supplementary File 1). This revealed core OTUs that were classified to *Corynebacterium durum* and *Eikenella* sp. in the supragingival plaque, and *Oribacterium sinus, Bulleidia moorei,* and *Campylobacter* sp. found as core OTUs jointly in the tongue plaque and saliva. Most of the core OTUs were shared between multiple sites, with relatively few found to be specific to one oral site.

### Patterns of temporal stability are dependent on time scale and dissimilarity measure

**Fig. 4:**
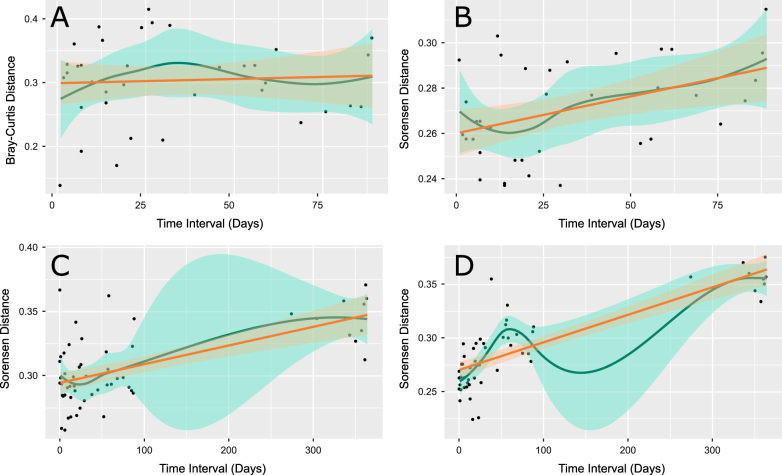
Lag plots of beta-diversity measures between sample pairs against the time elapsed between sample dates. *Yellow lines* are the linear least squares regression fits with 95% confidence bands and *blue lines* are the Loess locally weighted regression curves with 95% confidence bands. **a** Bray–Curtis distances between saliva samples from subject 7. **b** Sorensen distances between saliva samples from subject 7. **c** Sorensen distances between saliva samples from subject 1. **d** Sorensen distances between saliva samples from subject 10

We regressed the beta-diversity measures between pairs of samples against the time that elapsed between sample collection points in order to quantify the “drift” of the community composition over time. Of the 30 subject-site pairs, 30% saw a significant drift in the Bray–Curtis dissimilarity measure (a weighted measure) over time (as assessed by *F*-test on the linear regression). In contrast, 63% of subject-site pairs were associated with significant drift when using the Sorensen dissimilarity distance metric, which is an unweighted measure. Drift was most acute in the saliva, where 80% of subjects were associated with a significant increase in Sorensen distance over time. Of the subjects, 60 and 50% demonstrated significant drift in supragingival plaque and tongue plaque, respectively, under the Sorensen distance. Under Bray–Curtis distances, 20, 30, and 40% of subjects saw significant drift in the saliva, supragingival plaque, and tongue plaque, respectively. The saliva of one subject showed no significant increase in Bray–Curtis dissimilarity over time (Fig. 4a), whereas the same samples showed a statistically significant increase in the Sorensen dissimilarity over time (Fig. 4b).

The sampling time scale can greatly affect the interpretation of community drift. For example, the first 90 days of saliva from Subject 1 showed a statistically insignificant (*p* > 0.01) increase in sample dissimilarities over time (Fig. 4c), whereas Subject 10 had a very rapid and statistically significant shift in community structure over that same time period (Fig. 4d). Despite different initial rates of community change, both subjects saw a significant drift over the entire 365-day period (*p* < 0.001 by *F*-test). We also observed cases of statistically insignificant drift over 365 days despite significant drift over the initial 90-day sample period (data not shown). In the majority of cases, linear models based on the first 90 days consistently overestimated the drift over the course of a year, as there appear to be factors limiting the amount communities can drift over time.

## Discussion

Temporal community shifts of the oral biofilm consortia are poorly understood relative to that in other sites in the human gastrointestinal tract. Although surveillance of bacterial biomarkers in the mouth can be useful for early disease prediction, monitoring disease progression, and treatment compliance, this approach would be futile without a solid understanding of the normal baseline fluctuations of these markers in health. Because the mouth is exposed to the external environment and subjected to brushing, flossing, and nutrient intake, it can be expected that microbial turnover in saliva, tooth-associated and tongue-associated plaque would vary significantly, which in turn affects the reliable utility of plaque and saliva in risk assessment, diagnostic or prognostic applications. We discovered that significant community drift can occur over both short and long time scales, but the magnitude and significance of this drift varies between subjects. In spite of this drift, some features of the community composition remain sufficiently stable to allow for a high subject classification rate. We identified 26 core OTUs that classified within *Streptococcus, Fusobacterium, Haemophilus, Neisseria, Prevotella*, and *Rothia* genera, which were present across ≥95% of samples derived from saliva, tongue plaque, and dental plaque. Within-sample diversity levels were consistent between subjects in the tongue, but inter-individual differences were evident in supragingival plaque and saliva. The strongest separation of communities was observed between individuals (as assessed by MRPP), with the differences between the three sample types having a significant but smaller effect size. Inter-personal community differences and intra-personal temporal variation suggest that care must be taken when selecting biomarkers for diagnosis and prognosis. Thorough testing must be undertaken to ensure that potential biomarkers are robust and generalize well outside of the sample cohort.

Our results indicate that oral bacterial communities that inhabit supragingival plaque, tongue plaque, and saliva are clearly distinct from one another (Fig. 1). Teeth represent a non-shedding surface that enables the accumulation of distinct biofilms, in comparison to mucosal surfaces in the oral cavity. Furthermore, supragingival plaque is susceptible to daily variations in oral hygiene habits (e.g., tooth-brushing and flossing). The difference in biological and physical properties of the tongue dorsum and supragingival surface (Fig. 1) appears to be reflected in the distinctiveness of their corresponding microbial communities.^4^ The higher variation observed within PD values from the supragingival plaque could be due to differences in oral care routine between subjects or within subjects across time. Because tooth brushing and flossing affect the supragingival communities more than those in tongue papillary sites that provide refuge to microbes and contribute to the salivary microbiome, this daily disruption in the community could be providing an opportunity for new taxa to take hold in the environment, accounting for the increase in diversity over time.

Our observation that tongue plaque and salivary communities are highly similar corroborates previous findings.^23,24^ Community similarity between these two sites as assessed by a weighted measure (Bray–Curtis) suggests that the majority of the high-abundance OTUs found in the saliva were derived from the tongue. This is unsurprising because the tongue dorsum constitutes a large surface area containing a high biofilm biomass that is subjected to bacterial shedding and cellular desquamation.^25^ Due to the ease of collection and convenience of sampling, saliva is sometimes used for prognostic and diagnostic applications. In particular, bacterial biomarkers in saliva are being investigated for their use in treatment monitoring during oral diseases such as periodontitis and caries,^26–28^ and researchers are investigating the correlation between the salivary microbiota and diseases such as inflammatory bowel disease^29^ and pancreatic cancer.^30^

The higher OTU count and wider inter-subject variation in PD seen in saliva can be attributed to the saliva bathing other oral sites, such as the supragingival tooth surface, and picking up additional microbial taxa. Despite significant differences between subjects in mean PD values in the supragingival plaque and saliva (Fig. 2b), the amount of deviation from the mean was similar from person to person within all three sites. This could be useful in monitoring for the onset of oral diseases in at-risk patients, as alpha diversity has been shown to change with some disease states.^31^ A significant deviation from a subject’s established baseline alpha diversity could signal that intervention is required. An established baseline value is important, as our results show that alpha diversity measures vary significantly between individuals (Fig. 2b).

There are those who suggest that the oral microbiota in health are in a state of homeostasis,^4,32,33^ whereas others suggest that it is dynamic.^34,35^ Our results show that both of these statements can be true. The existence of a core microbiota (Fig. 3), especially one that spans different individuals, oral sites, and time, suggests some level of stability. Furthermore, these persistently present core taxa account for a very large proportion of the sequences, demonstrating that the most abundant organisms are stable (in terms of presence, though possibly not in relative proportion). The core microbiota we uncovered are mostly known commensals, and our results are consistent with previous studies.^13,24,36^ The core *Porphyromonas* sp. oral clone CW034 is related closely to *Porphyromonas catoniae,* both of which are found mainly in subjects with good oral health.^4,37^ The presence of *Bergeyella* strain 602D02 in the core microbiota is intriguing because these bacteria are uncultivated and typically associated with the canine oral microbiome.^38^ Because most previous studies had only a single sample site, individual, or time-point, our core set can be viewed as one of the most discriminating produced to this date.

Despite the high proportion of OTUs that are shared between every subject, our results show that “homeostasis” is a varied concept. Separation by subject is seen in the NMDS ordinations (Fig. 1) and validated by MRPP. Random-forest classification was able to identify OTUs that are unique to individuals or groups of individuals, and achieved high classification accuracy. After 1 year, we were able to identify the source subject with 83–100% accuracy, depending on the site. Zarco *et al.* ^34^ describe a “variable” microbiota in addition to the core microbiota, which differs from person to person as a result of host lifestyle and genetics. Our results suggest that these variable microorganisms persist within an individual over time, allowing a person to be identified by their unique microbial fingerprint even up to a year later. Even though it is thought that these variable organisms are largely functionally redundant,^21^ they could be very important considerations when assessing personalized disease risk.

It is clear from our results that the composition of the abundant microbiota changes much more slowly than that of the rare microbiota. When assessing drift by way of the weighted Bray–Curtis dissimilarity measure, only 30% of subject-site pairs showed a significant shift in the microbiota over time. Because Bray–Curtis weights OTU contributions by their abundance, this measure considers mainly the abundant microorganisms, the majority of which belong to the core set. In contrast, the unweighted Sorensen dissimilarity measure showed that 63% of subject-site pairs with significant drift. This measure treats all OTUs equally, regardless of abundance. This increased incidence of community drift under an unweighted measure suggests that the composition of the rare microorganisms is much more fluid relative to the abundant organisms, which is consistent with other reports.^39,40^ The significant drift observed in 80% of subjects’ salivary communities could be due to the saliva picking up and aggregating the differences from multiple sites in the oral cavity.

The rate of community drift was not consistent between subjects. Our results show that even in health, the stability of oral microbial communities should not be assumed. Moreover, our results highlight that stability is often (if not always) temporary. Stability over a shorter period does not guarantee long-term stability (Fig. 4c) and likewise rapid short-term change does not guarantee long-term variability. Our analysis of inter-personal variations (MRPP and random-forest classifications) shows that, despite the amount of observed drift, some personalized features are retained over time. This highly variable rate of change in community structure warrants further investigation to determine if rapid or continuous community drift can prevent or spur the onset of oral disease. These results also highlight the importance of longitudinal monitoring, as the microbiota can shift significantly in a matter of days or weeks, even in a healthy individual.

In the case of oral health, our data suggest that microbial communities have both stable and variable components. The community structures found at each oral site are consistent across different subjects and across time. Certain taxa are maintained across time within individuals, including highly abundant core OTUs that are shared with all other subjects. Alpha diversity was relatively stable within an individual over time while beta diversity measures were less clear. Stability existed in some individuals, but depended on the dissimilarity measure used and the time-scale considered. Variability was more common with an unweighted dissimilarity measure that does not penalize rare microorganisms. In the future, more focus should be put on the variable microbiota and to elucidate their contribution to health maintenance and transition into disease.

## Materials and methods

### Participant demographics and sample collection

Ten healthy subjects were recruited through the University of Toronto, Faculty of Dentistry as per guidelines by the Research Ethics Board (REB 30044). Informed consent to participate was obtained for all volunteers, who were students pursuing Undergraduate or Graduate degrees in the Faculty of Dentistry; subjects included six males and four females aged 22–29 years, all non-smokers, with one subject reporting antibiotic usage within the previous 3 months prior to sample collection (Table 1; Supplementary File 2). Average decayed, missing, and filled teeth (DMFT) index scores for subjects were indicated at 2.4. Five subjects reported a DMFT index of 0, two subjects an index of 1, and the final three subjects reported indices of 3, 6, and 13. All volunteers reported no past history of periodontitis; only two subjects reported having had clinically diagnosed, mild gingivitis while all were without periodontal disease at intake as clinically assessed at the Faculty of Dentistry, University of Toronto. The absence of periodontitis was clinically assessed on all patients through analysis of tissue inflammation by positive architecture, tissue consistency, and presence of stippling, bleeding on probing scores as well as assessment of the absence of evidence of periodontal destruction including analysis of probing depths, recession and clinical attachment loss. Subjects with systemic disease and/or those who had undergone a professional dental cleaning within the last 6 months were excluded from the study. Subjects were sampled daily for four consecutive days (days 1, 2, 3, and 4), weekly for 4 weeks (days 7, 14, 21, and 28), and monthly for two additional months (days 60 and 90). Four of the ten subjects were sampled after 365 days from the first sampling date.

**Table 1:** Summary of subject demographic information

Demographics, clinical parameters, and habits	Participants
Sex	Six males; four females
Age (years)	25.3 ± 3.1
Periodontal diagnosis	*n* = 0
Decayed, missing, filled teeth (DMFT) index	Mean: 2.4; Mode: 0; Min: 0; Max: 13
Smokers	*n* = 0
Drinkers	*n* = 0
Diabetes	*n* = 0
Dietary restrictions	None *n* = 7; Vegetarian *n* = 1; Lactose intolerant *n* = 1; Kosher *n* = 1
Daily medication use	*n* = 1
Antibiotic use in past 3 months	*n* = 1
Recreational drug use	*n* = 0
History of radiotherapy	*n* = 0
Intraoral applications	Fixed retainer *n* = 3

Collection of all oral samples were performed in the morning and participants were asked to refrain from tooth brushing, flossing or use of mouth rinses for 12 h prior to sample collection. Supragingival plaque was collected using six sterile 10-μl pipette tips (Axygen, CA, USA) from fascial surfaces of teeth 16, 11, 21, 26, 36, 31, 41, and 46, as well as interproximally from between teeth 17/16, 16/15, 12/11, 11/21, 21/22, 37/36, 36/35, 32/31, 31/41, 41/42, 45/46, and 46/47. Plaque from the tongue was collected by carrying out two midline scrapings along the dorsum of the tongue using a single sterile 10 μl inoculation loop (Simport Scientific Inc., Canada). Collection of all oral samples was performed by the same clinician. Following collection, all plaque samples from a given subject and sample location were immediately pooled into one tube containing 1 ml of sterile PBS (Sigma-Aldrich, MO, USA) on ice and then stored at −80 °C until further processing. Stimulated salivary samples were collected in pre-weighed sterile 50-ml conical tubes (Falcon; FroggaBio, ON, CA) and submerged in ice. Participants chewed on a piece of plastic paraffin film (Parafilm; 25 cm^2^) and swallowed saliva accumulated during the first 30 s. Following that, patients expectorated once every 30 s into the conical tube placed on ice for a period of 5 min, or until salivary contents reached a total of 5 ml. Salivary flow rate was calculated by dividing the total volume of saliva accumulated by the time required for collection (ml min^−1^). Following collection, saliva was distributed into aliquots using sterile 1.5-ml microcentrifuge tubes (Eppendorf; Axygen, CA, USA) and stored at −80 °C until further processing.

### DNA extraction and sequencing

DNA was extracted from 500 μl of plaque using the PowerSoil DNA Isolation Kit (MO BIO, CA, USA) according to the manufacturer’s instructions, with the exception of the following modifications. Each plaque sample was homogenized by 15 passages through a 3-ml syringe with an 18G × 1” needle prior to DNA extraction. Samples were divided in two tubes prior to adding Solution C3 (MO BIO) and subsequently mixed onto a single spin filter. Final elution of DNA was done using 200 μl of Solution C6. DNA concentrations were determined by UV spectrophotometry (Ultrospec 3000, Pharmacia Biotech) at a wavelength of 260 nm.

The V3–V4 regions of the 16S rRNA gene were amplified using bacterial primer 341F^41^ and universal primer 806R.^42^ Primers contained six-base index sequences for sample multiplexing as well as the Illumina adapters. Each polymerase chain reaction (PCR) mixture consisted of 2.5 μl of 10× *Taq* buffer (New England BioLabs, MA, USA), 0.05 μl of 100 μM forward primer, 0.5 μl of 10 μM reverse primer, 0.05 μl of 100 mM dNTPs, 15 μg of bovine serum albumin, 0.125 μl of *Taq* DNA polymerase (0.6 U l^-1^), 1 μl of template (1–20 ng), and nuclease-free water up to a final volume of 25 μl. Each reaction was prepared in triplicate. PCR was performed by denaturing samples at 95 °C for 30 s, followed by 30 cycles of denaturation at 95 °C for 15 s, annealing at 50 °C for 30 s, and extension at 68 °C for 30 s; the final heating step was done at 68° for 5 min. A MiSeq instrument (Illumina Inc., San Diego, CA, USA) with the MiSeq Reagent Kit v2 (Illumina Canada Inc, Saint John, NB, Canada) was used to produce 250-base paired-end reads.

### Sequence data processing

Sequence data were processed with the AXIOME version 2.0.4 pipeline.^43^ Paired-end sequences were assembled by PANDAseq version 2.8^44^ with a quality threshold of 0.9. Sequences were clustered at 97% identity using the UPARSE algorithm in the USEARCH software, version 7.0.1090.^45^ The minimum OTU size was set to 2, and USEARCH performed *de novo* chimera checking and removal at this stage. The most frequently observed sequence was selected as representative for each OTU. Representative sequences were classified with the naïve Bayesian classifier version 2.2^46^ of the Ribosomal Database Project (RDP), requiring an 80% posterior probability for classification at each taxonomic level. RDP was trained against the concatenated Greengenes August 2013 revision^47^ and CORE Oral January 28 2014 revision^48^ reference sets. QIIME version 1.9.0^49^ was used to create and rarefy the OTU table, generate sequence alignments with PyNast version 1.2.2,^50^ and construct a phylogenetic tree with FastTree version 2.1.3.^51^ Using this tree, we used QIIME to calculate Faith’s PD measures for each sample. Core microbiota and lag plots were created using R version 3.1.3.^52^ Significant differences in PD measures between groups of samples were detected using the non-parametric pairwise Wilcox test in R with false discovery rate correction. The significance of within-group similarity and between-group differences of Bray–Curtis distance measures were computed using the MRPP implementation available in the R vegan library version 2.2–1.^53^ A NMDS ordination plot based on Bray–Curtis distances was created using the vegan library in R. Subject classification was performed using the random-forest classifier implementation in the Scikit-learn Python package, version 0.15.2.^54^ For the first classification involving all samples, five-fold stratified cross-validation was used and classification was performed on 100 different splits of training and test sets. For the classification of the 1-year samples, only the samples from that time-point were held out of the training set. Each of the random forests contained 100 trees.

### Availability of data

Raw sequence data and metadata are available at the European Bioinformatics Institute ENA under project accession PRJEB11529.

## Electronic supplementary material

Supplementary File 1

Supplementary File 2

## References

[1] Pflughoeft KJ, Versalovic J (2012). Human microbiome in health and disease. Annu. Rev. Pathol. Mech. Dis.

[2] Consortium THMP (2012). Structure, function and diversity of the healthy human microbiome. Nature.

[3] Stearns J. C. *et al*. Bacterial biogeography of the human digestive tract. *Sci. Rep**.* **1**, 170 (2011). 10.1038/srep00170 PMC3240969 22355685

[4] Marsh, P. D., Martin, M. V., Lewis, M. A. & Williams, D. *Oral Microbiol.* (Elsevier Health Sciences, 2009).

[5] Simón-Soro A, Mira A (2015). Solving the etiology of dental caries. Trends Microbiol..

[6] Hajishengallis G, Lamont Rj (2012). Beyond the red complex and into more complexity: the polymicrobial synergy and dysbiosis (PSD) model of periodontal disease etiology. Mol. Oral Microbiol.

[7] Lazarevic V, Whiteson K, François P, Schrenzel J (2010). The salivary microbiome, assessed by a high-throughput and culture-independent approach. J. Integr. OMICS.

[8] Zhang S-M (2011). Bacterial diversity of subgingival plaque in 6 healthy Chinese individuals. Exp. Ther. Med.

[9] Jiang W (2015). The impact of various time intervals on the supragingival plaque dynamic core microbiome. PLoS One.

[10] David LA (2014). Host lifestyle affects human microbiota on daily timescales. Genome Biol..

[11] Caporaso JG (2011). Moving pictures of the human microbiome. Genome Biol..

[12] Mark Welch J. L. *et al*. Dynamics of tongue microbial communities with single-nucleotide resolution using oligotyping. *Front. Microbiol**.* **5, 568** (2014). 10.3389/fmicb.2014.00568 PMC4224128 25426106

[13] Zaura E, Keijser BJ, Huse SM, Crielaard W (2009). Defining the healthy ‘core microbiome’ of oral microbial communities. BMC Microbiol..

[14] Human Microbiome Project Consortium & others (2012). A framework for human microbiome research. Nature.

[15] Segata N (2012). Composition of the adult digestive tract bacterial microbiome based on seven mouth surfaces, tonsils, throat and stool samples. Genome Biol..

[16] Flores GE (2014). Temporal variability is a personalized feature of the human microbiome. Genome Biol..

[17] Costello EK (2009). Bacterial community variation in human body habitats across space and time. Science.

[18] Cameron SJS, Huws SA, Hegarty MJ, Smith DPM, Mur LAJ (2015). The human salivary microbiome exhibits temporal stability in bacterial diversity. FEMS Microbiol. Ecol..

[19] Belstrøm D (2016). Temporal stability of the salivary microbiota in oral health. PLoS One.

[20] Faith DP (1992). Conservation evaluation and phylogenetic diversity. Biol. Conserv..

[21] Turnbaugh PJ (2007). The human microbiome project. Nature.

[22] Shade A, Handelsman J (2012). Beyond the Venn diagram: the hunt for a core microbiome. Environ. Microbiol.

[23] Mager DL, Ximenez-Fyvie LA, Haffajee AD, Socransky SS (2003). Distribution of selected bacterial species on intraoral surfaces. J. Clin. Periodontol..

[24] Simón-Soro A (2013). Microbial geography of the oral cavity. J. Dent. Res..

[25] Gibbons RJ, Houte JV (1975). Bacterial adherence in oral microbial ecology. Annu. Rev. Microbiol..

[26] Guo L, Shi W (2013). Salivary biomarkers for caries risk assessment. J. Calif. Dent. Assoc.

[27] Yoshizawa JM (2013). Salivary biomarkers: toward future clinical and diagnostic utilities. Clin. Microbiol. Rev..

[28] Zhang L, Henson BS, Camargo PM, Wong DT (2009). The clinical value of salivary biomarkers for periodontal disease. Periodontol. 2000.

[29] Said HS (2014). Dysbiosis of salivary microbiota in inflammatory bowel disease and its association with oral immunological biomarkers. DNA Res..

[30] Torres PJ (2015). Characterization of the salivary microbiome in patients with pancreatic cancer. PeerJ.

[31] Abusleme L (2013). The subgingival microbiome in health and periodontitis and its relationship with community biomass and inflammation. ISME J..

[32] Ursell LK (2012). The interpersonal and intrapersonal diversity of human-associated microbiota in key body sites. J. Allergy Clin. Immunol..

[33] Darveau RP (2010). Periodontitis: a polymicrobial disruption of host homeostasis. Nat. Rev. Microbiol..

[34] Zarco M, Vess T, Ginsburg G (2012). The oral microbiome in health and disease and the potential impact on personalized dental medicine. Oral Dis.

[35] Xu X (2015). Oral cavity contains distinct niches with dynamic microbial communities. Environ. Microbiol..

[36] Jiang B. *et al*. Integrating next-generation sequencing and traditional tongue diagnosis to determine tongue coating microbiome. *Sci. Rep**.* **2, 936** (2012). 10.1038/srep00936 PMC3515809 23226834

[37] Sakamoto M (2015). Porphyromonas pasteri sp. nov., isolated from human saliva. Int. J. Syst. Evol. Microbiol.

[38] Dewhirst FE (2010). The human oral microbiome. J. Bacteriol..

[39] Lynch MDJ, Neufeld JD (2015). Ecology and exploration of the rare biosphere. Nat. Rev. Microbiol..

[40] Shade A (2014). Conditionally rare taxa disproportionately contribute to temporal changes in microbial diversity. mBio.

[41] Muyzer G, De Waal EC, Uitterlinden AG (1993). Profiling of complex microbial populations by denaturing gradient gel electrophoresis analysis of polymerase chain reaction-amplified genes coding for 16S rRNA. Appl. Environ. Microbiol..

[42] Caporaso JG (2011). Global patterns of 16S rRNA diversity at a depth of millions of sequences per sample. Proc. Natl Acad. Sci.

[43] Lynch MD, Masella AP, Hall MW, Bartram AK, Neufeld JD (2013). AXIOME: automated exploration of microbial diversity. GigaScience.

[44] Masella AP, Bartram AK, Truszkowski JM, Brown DG, Neufeld JD (2012). PANDAseq: paired-end assembler for illumina sequences. BMC Bioinform..

[45] Edgar RC (2013). UPARSE: highly accurate OTU sequences from microbial amplicon reads. Nat. Methods.

[46] Wang Q, Garrity GM, Tiedje JM, Cole JR (2007). Naive Bayesian classifier for rapid assignment of rRNA sequences into the new bacterial taxonomy. Appl. Environ. Microbiol..

[47] McDonald D (2012). An improved Greengenes taxonomy with explicit ranks for ecological and evolutionary analyses of bacteria and archaea. ISME J..

[48] Griffen AL (2011). CORE: a phylogenetically-curated 16S rDNA database of the core oral microbiome. PLoS One.

[49] Caporaso JG (2010). QIIME allows analysis of high-throughput community sequencing data. Nat. Methods.

[50] Caporaso JG (2010). PyNAST: a flexible tool for aligning sequences to a template alignment. Bioinformatics.

[51] Price MN, Dehal PS, Arkin AP (2010). FastTree 2–approximately maximum-likelihood trees for large alignments. PLoS One.

[52] R. Core Team. *R: A language and environment for statistical computing* (R Foundation for Statistical Computing, 2015).

[53] Oksanen J. *et al*. vegan: Community Ecology Package*.* (2016).

[54] Pedregosa F (2011). Scikit-learn: machine learning in python. J. Mach. Learn. Res..

